# Complete Genome Sequence of Enteroinvasive *Escherichia coli* O96:H19 Associated with a Severe Foodborne Outbreak

**DOI:** 10.1128/genomeA.00883-15

**Published:** 2015-08-06

**Authors:** Emily A. Pettengill, Maria Hoffmann, Rachel Binet, Richard J. Roberts, Justin Payne, Marc Allard, Valeria Michelacci, Fabio Minelli, Stefano Morabito

**Affiliations:** a Center for Food Safety and Applied Nutrition, Division of Microbiology, Office of Regulatory Science, U.S. Food and Drug Administration, College Park, Maryland, USA; b New England BioLabs, Inc., Ipswich, Massachusetts, USA; c EU Reference Laboratory for *Escherichia coli*, Department of Veterinary Public Health and Food Safety, Istituto Superiore di Sanità, Rome, Italy

## Abstract

We present here the complete genome sequence of a strain of enteroinvasive *Escherichia coli* O96:H19 from a severe foodborne outbreak in a canteen in Italy in 2014. The complete genome may provide important information about the acquired pathogenicity of this strain and the transition between commensal and pathogenic *E. coli*.

## GENOME ANNOUNCEMENT

Although enteroinvasive *Escherichia coli* (EIEC) is not recognized as the causal agent of severe foodborne outbreaks, a recent outbreak shows that EIEC can pose a serious public health threat to healthy individuals. EIEC strain O96:H19 was responsible for hospitalizing 32 people, with 109 total reported cases in 2014, which were traced to a canteen in a fire brigade in the city of Milan (1). The availability of the closed genome sequence provides an opportunity to investigate the pathogenicity of the organism and facilitate future outbreak tracking and faster identification. We announce the availability of the complete closed genome sequence of EIEC O96:H19.

Genomic DNA was isolated from overnight cultures grown at 37°C in Trypticase soy broth (Becton, Dickinson, NJ) and extracted using a DNeasy blood and tissue kit (Qiagen, Inc., Valencia, CA). The genome was sequenced using the Pacific Biosciences (PacBio) RS II sequencing platform. Size selection was performed with BluePippin (Sage Science, Beverly, MA), according to the manufacturer’s protocol, and the library was sequenced using the P6 chemistry on five single-molecule real-time (SMRT) cells (three with BluePippin and two without), with a 240-min collection protocol. Analysis of the sequence reads was implemented using SMRT Analysis 2.3.0. *De novo* assembly of the reads was performed using the PacBio HGAP.3 program, with default parameters. Overlapping regions identified at the end of the output assemblies (of chromosome and plasmids) were identified using Gepard (2) and trimmed, as previously described (3). Assemblies were annotated with the NCBI Prokaryotic Genomes Annotation Pipeline (PGAP) (http://www.ncbi.nlm.nih.gov/genomes/static/Pipeline.html) and subsequently deposited at DDBJ/EMBL/GenBank.

The chromosome consists of 4,947,513 bp (G+C content, 50.7%), with the invasion plasmid being 293,826 bp, and a smaller plasmid of 47,606 bp. Coverages for the genome and plasmids were 355×, 388×, and 61×, with 4,989, 370, and 57 genes, respectively. Sequencing of the strain also measured the kinetic variations (KVs) of nucleotide incorporation rates to infer DNA methyltransferase activities (4). The SMRT data of the methylome were analyzed and deposited in REBASE (5). Although the genome encoded seven putative DNA methyltransferases, only the classical *E. coli* M.Eco29787Dam and M.Eco29787Dcm DNA methyltransferases were active, based on the SMRT data.

### Nucleotide sequence accession numbers.

Complete genome and plasmid sequences have been deposited in DDBJ/EMBL/GenBank under the accession numbers CP011416 (chromosome), CP011417 (invasion plasmid), and CP011418 (additional plasmid).
